# Mortality and Hospital Stay Associated with Resistant *Staphylococcus aureus* and *Escherichia coli* Bacteremia: Estimating the Burden of Antibiotic Resistance in Europe

**DOI:** 10.1371/journal.pmed.1001104

**Published:** 2011-10-11

**Authors:** Marlieke E. A. de Kraker, Peter G. Davey, Hajo Grundmann, on behalf of the BURDEN study group

**Affiliations:** 1 Centre for Infectious Disease Control, National Institute for Public Health and the Environment (RIVM), Bilthoven, The Netherlands; 2 Department of Medical Microbiology, Academic Medical Centre Groningen, Groningen, The Netherlands; 3 Quality, Safety and Informatics Research Group, Dundee, United Kingdom; Brown University School of Medicine, United States of America

## Abstract

**Background:**

The relative importance of human diseases is conventionally assessed by cause-specific mortality, morbidity, and economic impact. Current estimates for infections caused by antibiotic-resistant bacteria are not sufficiently supported by quantitative empirical data. This study determined the excess number of deaths, bed-days, and hospital costs associated with blood stream infections (BSIs) caused by methicillin-resistant *Staphylococcus aureus* (MRSA) and third-generation cephalosporin-resistant *Escherichia coli* (G3CREC) in 31 countries that participated in the European Antimicrobial Resistance Surveillance System (EARSS).

**Methods and Findings:**

The number of BSIs caused by MRSA and G3CREC was extrapolated from EARSS prevalence data and national health care statistics. Prospective cohort studies, carried out in hospitals participating in EARSS in 2007, provided the parameters for estimating the excess 30-d mortality and hospital stay associated with BSIs caused by either MRSA or G3CREC. Hospital expenditure was derived from a publicly available cost model. Trends established by EARSS were used to determine the trajectories for MRSA and G3CREC prevalence until 2015. In 2007, 27,711 episodes of MRSA BSIs were associated with 5,503 excess deaths and 255,683 excess hospital days in the participating countries, whereas 15,183 episodes of G3CREC BSIs were associated with 2,712 excess deaths and 120,065 extra hospital days. The total costs attributable to excess hospital stays for MRSA and G3CREC BSIs were 44.0 and 18.1 million Euros (63.1 and 29.7 million international dollars), respectively. Based on prevailing trends, the number of BSIs caused by G3CREC is likely to rapidly increase, outnumbering the number of MRSA BSIs in the near future.

**Conclusions:**

Excess mortality associated with BSIs caused by MRSA and G3CREC is significant, and the prolongation of hospital stay imposes a considerable burden on health care systems. A foreseeable shift in the burden of antibiotic resistance from Gram-positive to Gram-negative infections will exacerbate this situation and is reason for concern.

*Please see later in the article for the Editors' Summary*

## Abstract 2

The authors calculate excess mortality, excess hospital stay, and related hospital expenditure associated with antibiotic-resistant bacterial bloodstream infections (*Staphylococcus aureus* and *Escherichia coli*) in Europe.

## Editors' Summary

**Background:**

Antimicrobial resistance—a consequence of the use and misuse of antimicrobial medicines—occurs when a microorganism becomes resistant (usually by mutation or acquiring a resistance gene) to an antimicrobial drug to which it was previously sensitive. Then standard treatments become ineffective, leading to persistent infections, which may spread to other people. With some notable exceptions such as TB, HIV, malaria, and gonorrhea, most of the disease burden attributable to antimicrobial resistance is caused by hospital-associated infections due to opportunistic bacterial pathogens. These bacteria often cause life-threatening or difficult-to-manage conditions such as deep tissue, wound, or bone infections, or infections of the lower respiratory tract, central nervous system, or blood stream. The two most frequent causes of blood stream infections encountered worldwide are *Staphylococcus aureus* and *Escherichia coli*.

**Why Was This Study Done?:**

Although hospital-associated infections have gained much attention over the past decade, the overall effect of this growing phenomenon on human health and medical services has still to be adequately quantified. The researchers proposed to fill this information gap by estimating the impact—morbidity, mortality, and demands on health care services—of antibiotic resistance in Europe for two types of resistant organisms that are typically associated with resistance to multiple classes of antibiotics and can be regarded as surrogate markers for multi-drug resistance—methicillin-resistant *S. aureus* and third-generation cephalosporin-resistant *E. coli*.

**What Did the Researchers Do and Find?:**

Recently, the Burden of Resistance and Disease in European Nations project collected representative data on the clinical impact of antimicrobial resistance throughout Europe. Using and combining this information with 2007 prevalence data from the European Antibiotic Resistance Surveillance System, the researchers calculated the burden of disease associated with methicillin-resistant *S. aureus* and third-generation cephalosporin-resistant *E. coli* blood stream infections. This burden of disease was expressed as excess number of deaths, excess number of days in hospital, and excess costs. Using statistical models, the researchers predicted trend-based resistance trajectories up to 2015 for the 31 participating countries in the European region.

The researchers included 1,293 hospitals from the 31 countries, typically covering 47% of all available acute care hospital beds in most countries, in their analysis. For *S. aureus*, the estimated number of blood stream infections totaled 108,434, of which 27,711 (25.6%) were methicillin-resistant. *E. coli* caused 163,476 blood stream infections, of which 15,183 (9.3%) were resistant to third-generation cephalosporins. An estimated 5,503 excess deaths were associated with blood stream infections caused by methicillin-resistant *S. aureus* (with the UK and France predicted to experience the highest excess mortality), and 2,712 excess deaths with blood stream infections caused by third-generation cephalosporin-resistant *E. coli* (predicted to be the highest in Turkey and the UK). The researchers also found that blood stream infections caused by both methicillin-resistant *S. aureus* and third-generation cephalosporin-resistant *E. coli* contributed respective excesses of 255,683 and 120,065 extra bed-days, accounting for an estimated extra cost of 62.0 million Euros (92.8 million international dollars). In their trend analysis, the researchers found that 97,000 resistant blood stream infections and 17,000 associated deaths could be expected in 2015, along with increases in the lengths of hospital stays and costs. Importantly, the researchers estimated that in the near future, the burden of disease associated with third-generation cephalosporin-resistant *E. coli* is likely to surpass that associated with methicillin-resistant *S. aureus*.

**What Do These Findings Mean?:**

These findings show that even though the blood stream infections studied represent only a fraction of the total burden of disease associated with antibiotic resistance, excess mortality associated with these infections caused by methicillin-resistant *S. aureus* and third-generation cephalosporin-resistant *E. coli* is high, and the associated prolonged length of stays in hospital imposes a considerable burden on health care systems in Europe. Importantly, a possible shift in the burden of antibiotic resistance from Gram-positive to Gram-negative infections is concerning. Such forecasts suggest that despite anticipated gains in the control of methicillin-resistant *S. aureus*, the increasing number of infections caused by third-generation cephalosporin-resistant Gram-negative pathogens, such as *E. coli*, is likely to outweigh this achievement soon. This increasing burden will have a big impact on already stretched health systems.

**Additional Information:**

Please access these websites via the online version of this summary at http://dx.doi.org/10.1371/journal.pmed.1001104.

- The World Health Organization has a fact sheet on general antimicrobial resistance
- The US Centers for Disease Control and Prevention webpage on antibiotic/antimicrobial resistance includes information on educational campaigns and resources
- The European Centre for Disease Control provides data about the prevalence of resistance in Europe through an interactive database

## Introduction

Managing increasingly limited resources is one of the key challenges in contemporary health care. Although antibiotic resistance is threatening the success of medical services, the exact societal implications have not been adequately quantified. In order to inform the public health debate in Europe and beyond, reliable estimates about excess mortality, morbidity, and costs are imperative. Data about this burden of disease (BoD) will enable evaluation of antimicrobial resistance (AMR) against other competing causes of morbidity and mortality. Moreover, medium-term trends can shed light on expected health care demands in the near future. Hitherto such information has not been available because of the absence of representative empirical data.

Recently, clinical studies carried out under the remit of the Burden of Resistance and Disease in European Nations (BURDEN) project [1] filled this void. Within the BURDEN framework, we estimated the impact of antibiotic resistance associated with the two most frequent causes of blood stream infections (BSIs) worldwide—*Staphylococcus aureus* and *Escherichia coli* [2]. For these pathogens we focused on two of the most clinically relevant resistance phenotypes—methicillin (meticillin) resistance for *S. aureus* and third-generation cephalosporin resistance for *E. coli*. Both phenotypes are typically associated with resistance to multiple classes of antibiotics and can be regarded as surrogate markers for multi-drug resistance. During these studies the clinical outcome and excess hospital stay for infected patients in 13 different hospitals in as many different European countries were prospectively ascertained [3],[4].

Here we provide estimates on the BoD of resistance by combining these results with prevalence data from the European Antibiotic Resistance Surveillance System (EARSS). EARSS data were collected under the supervision of two of the authors (H. G. and M. E. A. d. K.) at the Netherlands' National Institute for Public Health and the Environment between 1999 and 2009. We report excess mortality, excess hospital stay, and the related hospital expenditure associated with methicillin-resistant *S. aureus* (MRSA) and third-generation cephalosporin-resistant *E. coli* (G3CREC) bacteremias, and provide trend-based trajectories until 2015 for all countries that participated in EARSS in 2007.

## Methods

Analyses focused on episodes of MSRA and methicillin-susceptible *S. aureus* (MSSA), as well as G3CREC and third-generation cephalosporin-susceptible *E. coli* (G3SEC) BSIs reported to EARSS in 2007. In that year, 31 countries participated in EARSS, consisting of all European Union member states (excluding Slovakia), the two EU candidate countries (Croatia and Turkey), two European Free Trade Association (EFTA) countries (Norway and Iceland), and Israel, henceforth referred to as the European region. Susceptibility was determined according to consensus protocols published in the EARSS manual [5].

### Number and Incidence of Events

Since all diagnostic microbiological laboratories in Estonia, Hungary, Iceland, Ireland, Luxembourg, Malta, and Slovenia reported to EARSS (100% coverage), the total number of BSIs could be directly extracted from the EARSS database for these countries [6]. For the UK, the total number of BSIs caused by *S. aureus* (MRSA and MSSA) was provided by the Health Protection Agency, the Health Protection Scotland, the Welsh Healthcare Associated Infection Programme, and the Public Health Agency Northern Ireland through their mandatory reporting schemes. Data for the UK for *E. coli* (G3CREC and G3CSEC) were extracted from these programs' voluntary reporting schemes.

For all other countries, the expected total number of events was based on the number of bacteremias and hospital beds in the EARSS sample, combined with the total volume of acute care beds per country. National volume data was obtained from the online Eurostat database [7], the Organisation for Economic Co-operation and Development Health Data 2010 database [8], or provided by national institutes for public health (see Table S1 and Acknowledgments). Through the svyglm function in the R package “survey,” the odds of a BSI case per bed, including a 95% confidence interval (CI_95_), was estimated per BSI type and country. This method fits a generalized linear model with a quasi binomial distribution and logit link function. It accounts for the cluster effect (of sampling within countries) and finite populations (national volume of acute care beds). The model-derived odds, including CI_95_, were transformed to proportion (*P*) and then multiplied with the national volume of acute care beds to derive country-specific estimates for the total number of bacteremias (#BSIs). Resulting CI_95_ were smaller for countries with higher national EARSS coverage. Incidence was calculated by dividing these estimates, including the CI_95_, by population census data taken from the World Health Organization database [9].

### Excess Deaths, Bed-Days, and Costs

Adjusted odds ratios (aOR) for 30-d mortality, mortality proportions for the control group without *S. aureus* or *E. coli* bacteremia (*P*_0_), and excess length of stay in days (LOSR) were obtained from the clinical outcome studies described previously (Table S2) [3],[4]. The excess number of deaths (*D*) and extra bed-days (*B*) associated with BSIs caused by MRSA, MSSA, G3CREC, and G3CSEC were then calculated for each country, using Equation 1, derived from Bender and Blettner [10], and Equation 2, respectively:
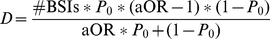
(1)

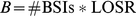
(2)


To determine CI_95_ for *D* and *B*, we used parametric bootstrapping. We sampled 10,000 simulations from the distribution for the log-odds of *P*, the log-odds of aOR, and the log-duration of LOSR. Thereafter, *D* and *B* were calculated for these sampled values, and CI_95_ could be based on the 2.5% and 97.5% quantiles from the resulting distributions. In this way, the CI_95_ included the uncertainty in the estimated number of bacteremias as well as the uncertainty in aOR and LOSR. As a result, CI_95_ were wider for countries with a low EARSS hospital participation ratio, and for BoD estimates for G3CREC compared to MRSA, because of wider confidence intervals for the clinical outcome measures for G3CREC (Table S2).

Excess hospital expenditure, including CI_95_, was based on the product of the excess number of bed-days and country-specific unit costs per hospital day. These hotel costs were derived from the WHO-CHOICE model [11],[12], and do not entail extra costs for procedures or treatments associated with antibiotic-resistant infections. The model-derived costs in local currency units of 2005 were indexed by country-specific consumer price indices [13]–[16] to approach costs in 2007. Hereafter these amounts were converted into Euros using historical exchange rates [17] and into international dollars using country-specific purchasing power parities for 2007 [12].

### Resistance Trajectories until 2015

The trajectories for BSIs caused by MRSA and G3CREC in the European region were based on trends in overall incidence of *S. aureus* and *E. coli* BSIs and changes in the relative proportions of MRSA and G3CREC as reported to EARSS. Data were extracted for all laboratories that consistently reported susceptibility results for *S. aureus* (1 January 2001–31 December 2009) and *E. coli* (1 January 2003–31 December 2009). The final results were generated in a multi-step procedure. First, the secular trends in the absolute number of *E. coli* and *S. aureus* BSIs until 2015 were obtained by linear regression. Second, the rate change in the proportions of BSIs with methicillin resistance and third-generation cephalosporin resistance was modeled by logistic regression [18]. In both models, year was included as a (log)linear independent variable. Higher order terms of time were included on the basis of the *F*-statistic and the likelihood ratio test (*p*<0.05). Model fit was assessed by the *F*- and Hosmer-Lemeshow statistic. The product of the two trends provided the crude trajectory for MRSA and G3CREC BSIs until 2015 for the sample of laboratories that consistently reported to EARSS. Finally, this trajectory was normalized against the reference incidence ascertained for 2007 and scaled to the total number of MRSA and G3CREC BSIs in the European region on the basis of total event estimates for 2007 (#BSIs) described above. All analyses were carried out using R 2.8.1 or SAS 9.2.

The Medical Ethics Committee of the University Medical Centre Utrecht waived ethical clearance for this study.

## Results

### Burden of Resistance

In 2007, 1,293 hospitals from 31 countries reported antimicrobial susceptibility test results for *S. aureus* and *E. coli* causing BSIs to the EARSS database [6]. At the national level, surveillance covered over 47% of all available acute care hospital beds for most countries (interquartile range 12%–99%). Altogether, susceptibility results for 18,000 *S. aureus* and 28,024 *E. coli* blood stream isolates were reported in 2007.

For *S. aureus*, the estimated number of BSIs in the European region totaled 108,434 (CI_95_ 103,637–112,948), of these, 27,711 (25.6%) were methicillin-resistant (range 0% in Iceland to 52% in Malta). *E. coli* caused 163,476 (CI_95_ 157,891–168,624) BSIs, of which 15,183 (9.3%) were resistant to third-generation cephalosporins (range 2% in Iceland to 40% in Turkey). The incidence of MRSA ranged from zero in Iceland and Norway to 18.7/100,000 (CI_95_ 17.6–19.9) in Portugal, and for G3CREC from 0.1 (reported number, no confidence interval) in Estonia to 8.1 (CI_95_ 7.0–9.2) in Israel (Table 1).

**Table 1:** Frequency of *S. aureus* and *E. coli* bacteremias in 31 European countries in 2007 by resistance phenotype.

Country	Number (CI_95_)				Incidence (CI_95_)			
	MRSA	MSSA	G3CREC	G3CSEC	MRSA	MSSA	G3CREC	G3CSEC
Austria	133 (132–134)	1,351 (1,348–1,354)	226 (225–228)	2,285 (2,281–2,289)	1.6 (1.6–1.6)	16.2 (16.1–16.2)	2.7 (2.7–2.7)	27.3 (27.3–27.4)
Belgium	250 (199–311)	836 (742–941)	84 (56–121)	1,987 (1,837–2,146)	2.4 (1.9–3)	8.0 (7.1–9.0)	0.8 (0.5–1.2)	19.0 (17.6–20.5)
Bulgaria	68 (41–107)	453 (375–544)	139 (98–192)	453 (374–543)	0.9 (0.5–1.4)	5.9 (4.9–7.1)	1.8 (1.3–2.5)	5.9 (4.9–7.1)
Croatia	215 (195–236)	351 (325–378)	38 (30–48)	1,228 (1,180–1,276)	4.7 (4.3–5.2)	7.7 (7.1–8.3)	0.8 (0.7–1.0)	27.0 (25.9–28)
Cyprus	87 (70–108)	94 (76–115)	45 (33–61)	187 (162–216)	10.2 (8.2–12.6)	11.0 (8.9–13.5)	5.3 (3.8–7.1)	21.9 (18.9–25.2)
Czech Republic	254 (241–269)	1,698 (1,662–1,735)	198 (186–210)	2,627 (2,583–2,671)	2.5 (2.4–2.6)	16.7 (16.3–17.0)	1.9 (1.8–2.1)	25.8 (25.4–26.2)
Denmark	21 (6–52)	1,544 (1,384–1,716)	81 (48–129)	2,590 (2,388–2,807)	0.4 (0.1–0.9)	28.4 (25.4–31.5)	1.5 (0.9–2.4)	47.6 (43.9–51.6)
Estonia^a^	18	188	2	217	1.3	14.1	0.1	16.3
Finland	12 (7–21)	576 (532–622)	32 (23–45)	1,572 (1,502–1,644)	0.2 (0.1–0.4)	10.9 (10.1–11.8)	0.6 (0.4–0.9)	29.8 (28.5–31.2)
France	4,523 (4,199–4,871)	12,860 (12,305–13,436)	617 (501–752)	30,029 (29,229–30,843)	7.3 (6.8–7.9)	20.9 (20.0–21.8)	1.0 (0.8–1.2)	48.7 (47.4–50)
Germany	2,484 (1,383–4,128)	12,311 (9,626–15,417)	1,921 (984–3,410)	21,632 (18,142–25,628)	3.0 (1.7–5)	14.9 (11.7–18.7)	2.3 (1.2–4.1)	26.2 (22–31)
Greece	1,207 (872–1,611)	1,334 (977–1,764)	110 (31–282)	1,270 (929–1,695)	10.8 (7.8–14.5)	12.0 (8.8–15.8)	1.0 (0.3–2.5)	11.4 (8.3–15.2)
Hungary^a^	279	920	59	1,120	2.8	9.2	0.6	11.2
Iceland^a^	0	65	4	101	0.0	21.6	1.3	33.6
Ireland^a^	507	825	88	1,663	11.8	19.2	2.0	38.7
Israel	573 (502–655)	1,168 (1,064–1,281)	558 (486–639)	3,415 (3,250–3,585)	8.3 (7.2–9.5)	16.9 (15.4–18.5)	8.1 (7.0–9.2)	49.3 (46.9–51.7)
Italy	2,679 (2,336–3,068)	5,177 (4,687–5,706)	1,149 (928–1,415)	9,264 (8,609–9,944)	4.6 (4.0–5.2)	8.8 (8.0–9.7)	2.0 (1.6–2.4)	15.7 (14.6–16.9)
Latvia	29 (19–41)	311 (279–347)	23 (15–34)	131 (110–155)	1.3 (0.9–1.8)	13.7 (12.2–15.2)	1.0 (0.6–1.5)	5.7 (4.8–6.8)
Lithuania	35 (25–47)	351 (317–387)	27 (19–38)	351 (317–388)	1.0 (0.7–1.4)	10.4 (9.4–11.4)	0.8 (0.6–1.1)	10.4 (9.4–11.4)
Luxembourg^a^	24	91	11	264	5.1	19.5	2.4	56.5
Malta^a^	55	50	15	102	13.5	12.3	3.7	25.1
Netherlands	36 (11–92)	2,985 (2,672–3,319)	205 (131–309)	4,734 (4,345–5,141)	0.2 (0.1–0.6)	18.2 (16.3–20.2)	1.2 (0.8–1.9)	28.8 (26.5–31.3)
Norway	0	1,280 (1,176–1,391)	59 (38–86)	2,814 (2,671–2,961)	0.0	27.3 (25.0–29.6)	1.3 (0.8–1.8)	59.9 (56.8–63)
Poland	322 (220–449)	1,777 (1,520–2,058)	64 (25–133)	2,775 (2,452–3,122)	0.8 (0.6–1.2)	4.7 (4.0–5.4)	0.2 (0.1–0.3)	7.3 (6.4–8.2)
Portugal	1,989 (1,870–2,113)	2,159 (2,037–2,287)	431 (376–492)	3,866 (3,707–4,030)	18.7 (17.6–19.9)	20.3 (19.2–21.5)	4.1 (3.5–4.6)	36.4 (34.9–37.9)
Romania	139 (76–233)	382 (268–529)	225 (141–344)	551 (413–720)	0.6 (0.4–1.1)	1.8 (1.3–2.5)	1.0 (0.7–1.6)	2.6 (1.9–3.4)
Slovenia^a^	36	394	35	832	1.8 (1.7–1.9)	19.7 (19.4–19.9)	1.7 (1.7–1.8)	41.5 (41.2–41.9)
Spain	2,223 (1,979–2,484)	6,663 (6,244–7,110)	1,385 (1,195–1,595)	18,405 (17,725–19,096)	5.0 (4.5–5.6)	15.0 (14.1–16.1)	3.1 (2.7–3.6)	41.6 (40–43.1)
Sweden	25 (20–31)	2,533 (2,482–2,585)	90 (80–101)	4,382 (4,316–4,446)	0.3 (0.2–0.3)	27.8 (27.2–28.3)	1.0 (0.9–1.1)	48.1 (47.3–48.8)
Turkey	3,968 (3,599–4,349)	7,677 (7,178–8,202)	4,440 (4,051–4,849)	6,666 (6,187–7,163)	5.3 (4.8–5.8)	10.3 (9.6–11.0)	5.9 (5.4–6.5)	8.9 (8.3–9.6)
UK^a^	5,520	11,021	2,821	20,779	9.1	20.3	4.6	34.2
Total number/overall incidence	27,711 (26,042–29,103)	80,723 (77,595–83,846)	15,183 (13,852–16,353)	148,292 (144,039–152,271)	4.8 (4.5–5.0)	13.9 (13.4–14.5)	2.6 (2.4–2.8)	25.6 (24.9–26.3)

Estimated absolute numbers and the incidence expressed per 100,000 individuals of MRSA, MSSA, G3CREC, and G3CSEC. Countries include all European Union member states (excluding Slovakia), both EU candidate countries (Croatia and Turkey), two EFTA countries (Iceland and Norway), and Israel.

a Total number and incidence of bacteremias as reported to EARSS or by national health authorities (UK), no confidence intervals provided.

In the same year, an estimated 5,503 (CI_95_ 3,136–8,267) excess deaths were associated with BSIs caused by MRSA and 2,712 (CI_95_ 595–5,780) with BSIs caused by G3CREC, based on risk estimates from previous clinical outcome studies (Table S2) [3],[4]. In 2007, the UK and France predictably experienced the highest excess mortality associated with BSIs caused by MRSA, with 1,096 (CI_95_ 627–1,650) and 898 (CI_95_ 511–1,364) fatalities, respectively. For BSIs caused by G3CREC, excess mortality was predicted to be the highest in Turkey (793, CI_95_ 178–1,716) and the UK (504, CI_95_ 114–1,078) and lowest in Iceland (1, CI_95_ 0–2) and Estonia (0, CI_95_ 0–1) (Table S3).

At the same time, BSIs caused by MRSA and G3CREC contributed an excess of 255,683 (CI_95_ 142,934–375,880) and 120,065 (CI_95_ 52,272–198,338) extra bed-days. This excess length of hospital stay accounted for an estimated extra cost of 62.0 million Euros (CI_95_ 31.4–100.0 million), equivalent to 92.8 million international dollars (CI_95_ 47.0–149.0 million) in 2007 (Table S4).

### Trends and Future Trajectories

For *S. aureus*, 266 laboratories serving 810 hospitals in 25 countries consistently reported antibiotic susceptibility test results to the EARSS database between 1 January 2001 and 31 December 2009, totaling 121,469 blood isolates. During the same period, the absolute number of reported *S. aureus* BSIs increased from 10,874 to 15,299. Methicillin resistance increased from 19.1% in 2001 to 22.6% in 2005 and then decreased to 18.0% by 2009 (Figure S1).

For *E. coli*, 281 laboratories serving 791 hospitals in 28 countries consistently reported antimicrobial susceptibility (136,217 blood isolates) between 1 January 2003 and 31 December 2009. During this time, the number of *E. coli* BSIs increased from 19,332 to 29,938. Resistance to G3CEP increased from 2.7% in 2003 to 8.2% in 2009 (Figure S2).

**Figure 1:**
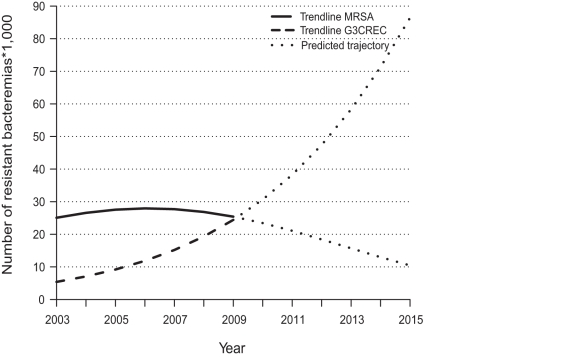
Trends in the estimated number of MRSA and G3CREC bacteremias in the European region. Extrapolated EARSS numbers for 2003–2009, and future trajectories based on regression analysis for 2010–2015.

Figure 1 shows that, based on the relative trends from EARSS, the number of G3CREC bacteremias is likely to surpass the number of MRSA bacteremias in the near future. As a result, the additive burden of G3CREC and MRSA bacteremias in the European region will increase. If current trends prevail, the trajectories suggest that about 97,000 resistant BSIs and 17,000 associated fatalities could be expected in 2015. Hospital stay and expenditure would likewise increase.

## Discussion

By combining representative data on clinical outcome with population-based incidence figures, we estimated that more than 8,000 deaths and 62 million Euros in excess costs were associated with MRSA and G3CREC BSIs in the European region in 2007. To our knowledge, this is the first quantification of the BoD for antibiotic resistance in this region based on empirical data.

As early as 1998, an EU conference titled “The Microbial Threat” in Visby, Denmark, emphasized that the most important questions regarding increasing resistance concern the potential rise in morbidity, mortality, and costs [19]. Although more insight into the prevalence of antibiotic resistance has been gained [20]–[23], its overall effect on human health and societies remained to be defined. With some notable exceptions such as TB, HIV, malaria and gonorrhea, most of the disease burden attributable to AMR is caused by hospital-associated infections due to opportunistic bacterial pathogens. These often cause life-threatening or difficult-to-manage conditions such as deep tissue, wound, or bone infections or infections of the lower respiratory tract, central nervous system, or the bloodstream. We chose to investigate the BoD associated with AMR in BSIs. This decision was guided by the clinical importance of BSIs and the fact that prevalence data are available from one of the largest international surveillance systems (EARSS), recording antibiotic resistance for more than 1,400 European hospitals.

This study took advantage of recently published observational studies [3],[4]. These were purposely designed to provide an objective measure about excess mortality and length of hospital stay associated with MRSA and G3CREC BSIs for Europe. In addition, these studies took into account that MRSA and G3CREC bacteremias add to, rather than replace, the BoD caused by their susceptible counterparts [24]–[28]. To this effect, clinical outcome measures of patients with resistant as well as susceptible BSIs were compared to those of uninfected controls.

However, three potential threats to the validity of our estimates need to be considered. First, a potential source of bias is inherent to the surveillance data from EARSS. Second, bias may have been introduced through the clinical outcome measures from the BURDEN studies. Finally, effect modification due to varying levels of appropriate empirical treatment could have influenced our results.

The incidence of resistant BSIs reported by EARSS hospitals may differ from the national average, because of the size, different standards of care, and/or different local epidemiology of EARSS hospitals [29],[30]. This limitation in representativeness, however, only applies to a minority of countries. In 2007, ten of the EARSS national networks collected complete data for all acute care hospitals in these countries, a further 11 networks had coverage above 50% [6]. Incidence data from alternative sources, such as the BMR-Raisin network in France [31] and nationwide registration of *S. aureus* BSIs in Denmark [32], underline the representativeness of our estimates for these countries. However, for Germany, Italy, and Greece, where EARSS population coverage was below 20%, the estimated incidence may be less reliable. The direction of this potential bias is not easily predictable. In the case of Germany, where mandatory reporting for MRSA bacteremia started in 2009, data indicate that our model may have underestimated the true burden [33].

Compared to the clinical outcome studies that provided the baseline for the current investigation [3],[4], other recent, well-designed studies came to more conservative estimates for the clinical impact of MRSA [34],[35]. However, differences in study design and outcome measures make direct comparisons difficult; presented hazard ratios [34],[35] cannot be directly compared with odds ratios [3],[4]. Moreover, Wolkewitz et al. [34] focused on a single center, while Lambert et al. [35] observed outcomes of patients during their stay in intensive care units. Estimates used in the present study were based on 30-d follow-up in multiple centers from different European countries. This provided better comprehensiveness and consequently bears more relevance to the BoD estimates for acute care for Europe as a whole.

Finally, the current analysis did not consider the impact of a delay in appropriate therapy. This may have had a negative effect on clinical outcome. However, considering that ineffective empirical therapy is often a direct consequence of antibiotic resistance, we did not separate this effect from our analysis as we regard it as an integral part of the burden caused by resistance.

Using empirical data improved the validity of our estimates of the impact of antibiotic resistance in the European region. However, limiting our study to BSIs caused by two, albeit important, pathogens ignores the consequences of antibiotic resistance due to infections of other causes and at other anatomical sites. More work is therefore required to fathom the total magnitude of antibiotic resistance as a public health issue. A recent report from the European Centre for Disease Control [36] also estimated the human and economic BoD of antibiotic resistance in Europe. This report included six of the bacterial pathogens under surveillance by EARSS and, in addition to BSIs, included four other types of infections: pneumonia, and abdominal, urinary tract, and soft tissue infections. It should be noted that these estimates must be used with caution for several reasons. Country-specific incidence estimates for BSIs were extrapolated from self-reported national catchment populations, an approach which frequently overestimates EARSS coverage [6]. In addition, BoD estimates were mainly based on risk data from small, single center studies. At the same time, questionable assumptions were necessary to estimate the incidence and BoD for infections at other anatomical sites based on estimates for BSIs.

Even when considering these restrictions, our results suggest that mortality attributed to AMR is considerable, but not excessive when compared to other causes. For high income countries in Europe, including 21 of 31 participating in EARSS, the World Health Organization reports that the highest number of deaths is associated with cardiovascular disorders (373 deaths per 100,000) [37]. Among communicable diseases, lower respiratory tract infections (29.5 per 100,000) rank highest [37]. For G3REC and MRSA BSIs, the estimated mortality (1.5 per 100,000 in the high income countries) is on par with rates for HIV/AIDS (1.5 per 100,000) or tuberculosis (1.0 per 100,000) [37].

How will this change in the near future? Here, we project that the combined burden of resistance of MRSA and G3CREC will be growing, leading to a predicted incidence of 3.3 associated deaths per 100,000 inhabitants in 2015. The burden of resistance will thereby likely surpass the current estimates for casualties associated with, for example, cervical cancer (2.7 per 100,000) [37]. Although these presented forecasts emphasize the increasing importance of resistance, especially for third-generation cephalosporin resistance in *E. coli* BSIs, these predictions should be interpreted with caution. The presented trajectories were based on a continuation of current trends, reflecting the unlikely scenario that saturation effects of present control efforts [38] or expansion of newly emerging clones or resistance mechanisms [39],[40] will not take place. Since these events are highly unpredictable, they may thwart attempts towards reliable trend analysis.

We conclude that excess mortality associated with MRSA and G3CREC is high, even though it represents only a fraction of the total BoD associated with antibiotic resistance. Forecasts about changes in the coming years are disturbing; despite anticipated gains in the control of MRSA, the persistently increasing number of infections caused by third-generation cephalosporin-resistant Gram-negative pathogens is likely to outweigh this achievement soon.

## Supporting Information

Figure S1 **Trends in the number of** ***S. aureus*** **BSIs and the proportion of these that were resistant for methicillin for EARSS laboratories consistently reporting from 2001–2015.** (A) Number of *S. aureus* BSIs. (B) Proportion resistant for methicillin. Diamonds indicate ascertained values, and trend line projections are based on regression analysis; regression equations are included.
(PDF)

Figure S2 **Trends in the number of** ***E. coli*** **BSIs and the proportion of these that were resistant for third-generation cephalosporins for EARSS laboratories consistently reporting from 2003–2015.** (A) Number of *E. coli* BSIs. (B) Proportion resistant for third-generation cephalosporins. Diamonds indicate ascertained values, and trend line projections are based on regression analysis; regression equations are included.
(PDF)

Table S1 **Number of acute care beds for 2007.** Beds per 10,000 inhabitants.
(PDF)

Table S2 **Parameter estimates.** Adjusted odds ratios for 30-d mortality and excess length of hospital stay, in days, associated with MRSA, MSSA, G3CREC, and G3CSEC bacteremias, and the derived number needed to be exposed for one excess death (NNE) [3],[4].
(PDF)

Table S3 **Estimated number of excess deaths associated with MRSA, MSSA, G3CREC, and G3CSEC bacteremias in 2007.** Countries include all European Union member states (excluding Slovakia), both candidate countries (Croatia and Turkey), two EFTA countries (Iceland and Norway), and Israel.
(PDF)

Table S4 **Estimated excess number of bed-days and costs associated with MRSA, MSSA, G3CREC, and G3CSEC bacteremias in 2007.** Countries include all European Union member states (excluding Slovakia), both candidate countries (Croatia and Turkey), two EFTA countries (Iceland and Norway), and Israel.
(PDF)

## Abbreviations

AMR: antimicrobial resistance
BoD: burden of disease
BSI: blood stream infection
BURDEN: Burden of Resistance and Disease in European Nations
CI_95_: 95% confidence interval
EARSS: European Antimicrobial Resistance Surveillance System
EFTA: European Free Trade Association
G3CREC: third-generation cephalosporin-resistant *Escherichia coli*
G3SEC: third-generation cephalosporin-susceptible *E. coli*
MRSA: methicillin-resistant *Staphylococcus aureus*
MSSA: methicillin-susceptible *Staphylococcus aureus*
