# Supplementary material for: Mortality and Hospital Stay Associated with Resistant Staphylococcus aureus and Escherichia coli Bacteremia: Estimating the Burden of Antibiotic Resistance in Europe
Source: PLoS Med. 2011 Oct 11;8(10):e1001104. doi: 10.1371/journal.pmed.1001104 (PMC3191157; doi:10.1371/journal.pmed.1001104)
Supplement: Figure S1 — Trends in the number of S. aureus BSIs and the proportion of these that were resistant for methicillin for EARSS laboratories consistently reporting from 2001–2015. (A) Number of S. aureus BSIs. (B) Proportion resistant for methicillin. Diamonds indicate ascertained values, and trend line projections are based on regression analysis; regression equations are included. (PDF) [file pmed.1001104.s001.pdf]

Figure S1 Trends in the number of S. aureus BSIs and the proportion of these that were resistant for methicillin for EARSS laboratories consistently reporting from 2001-2015. (A) Number of S. aureus BSIs. (B) Proportion resistant for methicillin. Diamonds indicate ascertained values, and trendline projections are based on regression analysis; regression equations are included.

A

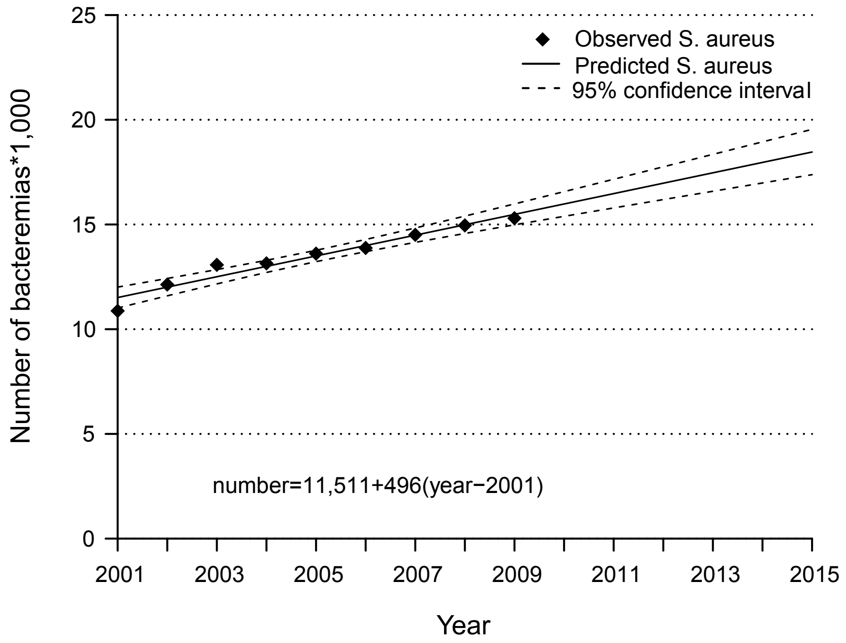

B

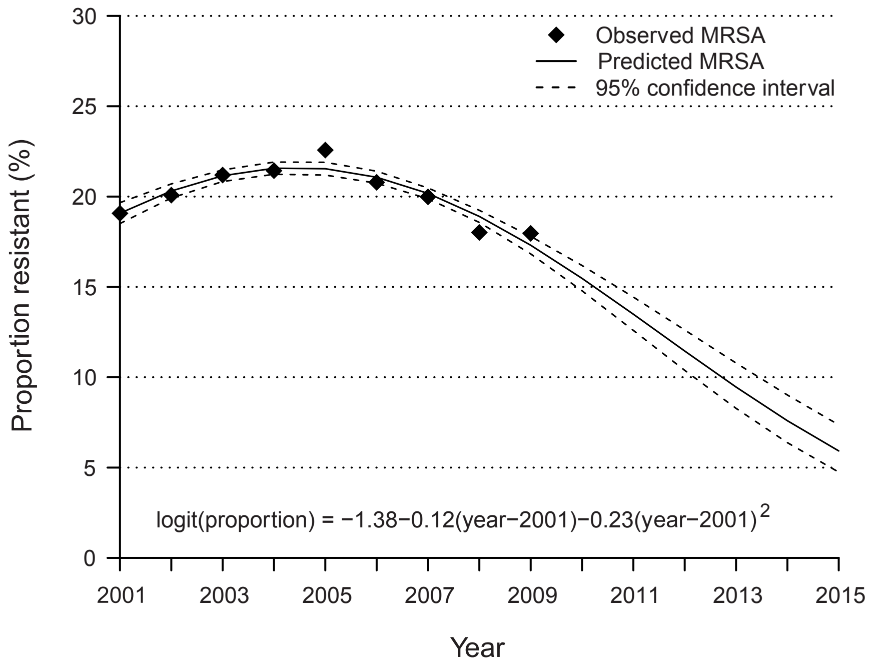

de Kraker MEA et al. Burden of Resistant Bacteremias in Europe (2011) PLoS Medicine
