# Supplementary material for: Mortality and Hospital Stay Associated with Resistant Staphylococcus aureus and Escherichia coli Bacteremia: Estimating the Burden of Antibiotic Resistance in Europe
Source: PLoS Med. 2011 Oct 11;8(10):e1001104. doi: 10.1371/journal.pmed.1001104 (PMC3191157; doi:10.1371/journal.pmed.1001104)
Supplement: Figure S2 — Trends in the number of E. coli BSIs and the proportion of these that were resistant for third-generation cephalosporins for EARSS laboratories consistently reporting from 2003–2015. (A) Number of E. coli BSIs. (B) Proportion resistant for third-generation cephalosporins. Diamonds indicate ascertained values, and trend line projections are based on regression analysis; regression equations are included. (PDF) [file pmed.1001104.s002.pdf]

Figure S2 Trends in the number of E. coli BSIs and the proportion of these that were resistant for third-generation cephalosporins for EARSS laboratories consistently reporting from 2003-2015. (A) Number of E. coli BSIs. (B) Proportion resistant for third-generation cephalosporins. Diamonds indicate ascertained values, and trendline projections are based on regression analysis; regression equations are included.

A

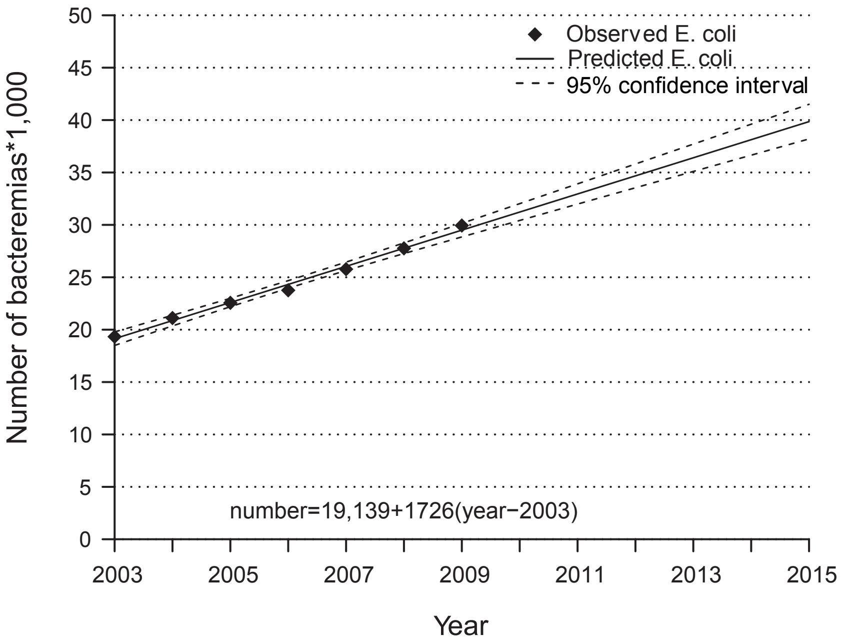

B

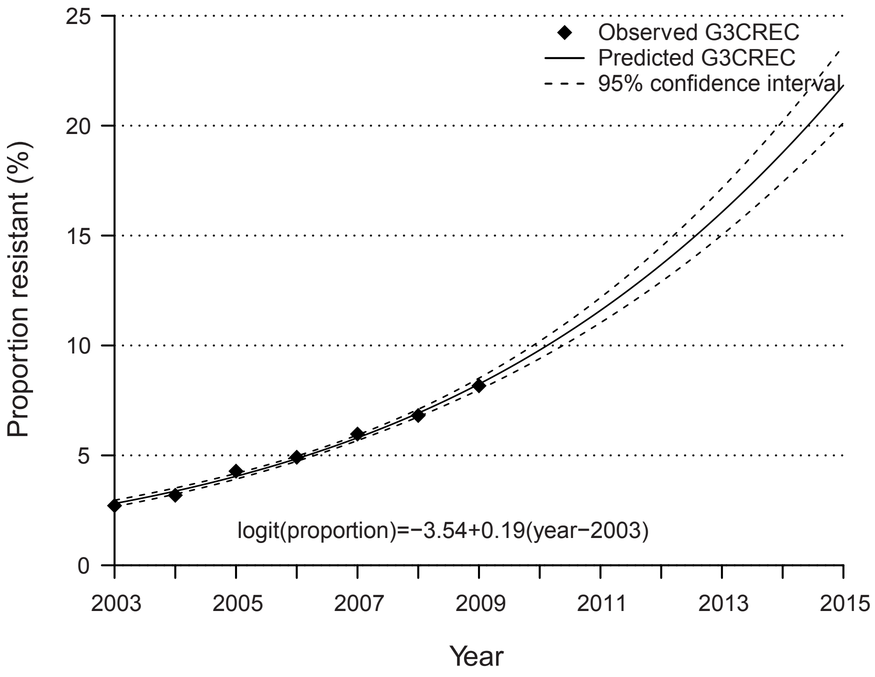

de Kraker MEA et al. Burden of Resistant Bacteremias in Europe (2011) PLoS Medicine
