# Supplementary material for: Mortality and Hospital Stay Associated with Resistant Staphylococcus aureus and Escherichia coli Bacteremia: Estimating the Burden of Antibiotic Resistance in Europe
Source: PLoS Med. 2011 Oct 11;8(10):e1001104. doi: 10.1371/journal.pmed.1001104 (PMC3191157; doi:10.1371/journal.pmed.1001104)
Supplement: Table S1 — Number of acute care beds for 2007. Beds per 10,000 inhabitants. (PDF) [file pmed.1001104.s003.pdf]

Table S1. Number of acute care beds for 2007: beds per 10,000 inhabitants.

Country	Number of hospital beds (per 10,000 inhabitants)*
Austria	56.8
Belgium	43.0
Bulgaria	49.1
Croatia	34.0
Cyprus	34.8
Czech Republic	51.5
Denmark	30.8
Estonia	38.0
Finland	20.5
France	35.4
Germany	56.9
Greece	39.6
Hungary	41.4
Iceland	40.7
Ireland	26.7
Israel	21.1
Italy	31.4
Latvia	52.4
Lithuania	51.1
Luxembourg	44.4
Malta	26.9
Netherlands	28.9
Norway	27.6
Poland	46.2
Portugal	27.9
Romania	44.8
Slovenia	37.8
Spain	25.6
Sweden	23.2
Turkey	23.7
United Kingdom	27.3

* All data were provided by Eurostat [7], except for Finland, National Public Health Institute (KTL); Iceland, Ministry of Health Iceland; Israel, Ministry of Health Israel; and Sweden, Swedish Institute for Infectious Disease Control.

Reference

7. Eurostat (2010) Statistics Database [database]. Available: http://epp.eurostat.ec.europa.eu/portal/page/portal/eurostat/home. Accessed August 1, 2010.
