# Supplementary material for: Mortality and Hospital Stay Associated with Resistant Staphylococcus aureus and Escherichia coli Bacteremia: Estimating the Burden of Antibiotic Resistance in Europe
Source: PLoS Med. 2011 Oct 11;8(10):e1001104. doi: 10.1371/journal.pmed.1001104 (PMC3191157; doi:10.1371/journal.pmed.1001104)
Supplement: Table S2 — Parameter estimates. Adjusted odds ratios for 30-d mortality and excess length of hospital stay, in days, associated with MRSA, MSSA, G3CREC, and G3CSEC bacteremias, and the derived number needed to be exposed for one excess death (NNE) [3],[4]. (PDF) [file pmed.1001104.s004.pdf]

Table S2. Parameter estimates: Adjusted odds ratios for 30-day mortality and excess length of hospital stay, in days, associated with methicillin-resistant (MRSA) and methicillin-susceptible (MSSA) *S. aureus* and third-generation cephalosporin-resistant (G3CREC) and cephalosporin-susceptible (G3CSEC) *E. coli* bacteremias, and the derived number needed to be exposed for one excess death (NNE) [3,4].

Bacteremias	Adjusted odds ratio for 30-day mortality (CI_{95})	NNE (CI_{95})	Excess length of hospital stay in days (CI_{95})
MRSA	4.4 (2.8-7.0)	4.9 (3.4-9.2)	9.2 (5.2-13.5)
MSSA	2.4 (1.7-3.3)	11.2 (7.4-20.0)	8.6 (6.8-10.4)
G3CREC	4.6 (1.7-12.3)	6.3 (2.6-25.2)	7.9 (3.5-13.0)
G3CSEC	1.9 (1.4-2.5)	18.9 (11.1-35.7)	2.9 (1.7-4.0)

CI_{95}= 95% confidence interval

References

3. De Kraker MEA, Wolkewitz M, Davey PG, Koller W, Berger J, et al. (2011) Clinical impact of antimicrobial resistance in European hospitals: excess mortality and length of hospital stay related to methicillin-resistant *Staphylococcus aureus* bloodstream infections. Antimicrob Agents Chemother 55: 1598-1605.
4. De Kraker MEA, Wolkewitz M, Davey PG, Koller W, Berger J, et al. (2011) Burden of antimicrobial resistance in European hospitals: excess mortality and length of hospital stay associated with bloodstream infections due to *Escherichia coli* resistant to third-generation cephalosporins. J Antimicrob Chemother 66: 398-407.
