# Supplementary material for: Mortality and Hospital Stay Associated with Resistant Staphylococcus aureus and Escherichia coli Bacteremia: Estimating the Burden of Antibiotic Resistance in Europe
Source: PLoS Med. 2011 Oct 11;8(10):e1001104. doi: 10.1371/journal.pmed.1001104 (PMC3191157; doi:10.1371/journal.pmed.1001104)
Supplement: Table S4 — Estimated excess number of bed-days and costs associated with MRSA, MSSA, G3CREC, and G3CSEC bacteremias in 2007. Countries include all European Union member states (excluding Slovakia), both candidate countries (Croatia and Turkey), two EFTA countries (Iceland and Norway), and Israel. (PDF) [file pmed.1001104.s006.pdf]

**Table S4. Estimated excess number of bed-days and costs associated with methicillin-resistant (MRSA) and methicillin-susceptible (MSSA) *S. aureus* and third-generation cephalosporin-resistant (G3CREC) and third-generation cephalosporin-susceptible (G3CSEC) *E. coli* bacteremias in 2007.**

| Country | Excess number of bed-days in thousands ($CI_{95}$) | | | | Excess costs in thousand Euros ($CI_{95}$) | | | |
|---|---|---|---|---|---|---|---|---|
| | MRSA | MSSA | G3CREC | G3CSEC | MRSA | MSSA | G3CREC | G3CSEC |
| Austria | 1.2 (0.7-1.8) | 11.6 (9.2-14.1) | 1.8 (0.8-3.0) | 6.6 (4.0-9.2) | 279 (157-413) | 2630 (2092-3192) | 407 (180-674) | 1492 (909-2083) |
| Belgium | 2.3 (1.2-3.6) | 7.2 (5.5-8.9) | 0.7 (0.3-1.2) | 5.7 (3.5-8.0) | 506 (273-787) | 1575 (1218-1967) | 147 (59-269) | 1255 (762-1764) |
| Bulgaria | 0.6 (0.3-1.1) | 3.9 (2.9-5.1) | 1.1 (0.5-2.0) | 1.3 (0.8-1.9) | 20 (9-37) | 127 (94-165) | 36 (15-65) | 42 (25-62) |
| Croatia | 2.0 (1.1-3.0) | 3.0 (2.4-3.7) | 0.3 (0.1-0.5) | 3.5 (2.2-4.9) | 136 (76-204) | 207 (163-254) | 21 (9-36) | 243 (148-340) |
| Cyprus | 0.8 (0.4-1.3) | 0.8 (0.6-1.1) | 0.4 (0.1-0.6) | 0.5 (0.3-0.8) | 119 (64-185) | 118 (87-156) | 52 (22-94) | 79 (47-115) |
| Czech Rep. | 2.3 (1.3-3.5) | 14.5 (11.6-17.6) | 1.6 (0.7-2.6) | 7.5 (4.6-10.5) | 214 (120-319) | 1329 (1060-1612) | 143 (63-237) | 690 (420-963) |
| Denmark | 0.2 (0.1-0.5) | 13.2 (10.3-16.4) | 0.6 (0.2-1.3) | 7.4 (4.5-10.5) | 55 (15-143) | 3745 (2906-4642) | 182 (69-354) | 2108 (1283-2978) |
| Estonia[a] | 0.2 (0.1-0.2) | 1.6 (1.3-2.0) | 0.0 | 0.6 (0.4-0.9) | 13 (8-20) | 130 (103-158) | 1 (1-2) | 50 (31-70) |
| Finland | 0.1 (0.0-0.2) | 4.9 (3.9-6.1) | 0.3 (0.1-0.5) | 4.5 (2.8-6.3) | 27 (12-50) | 1144 (901-1407) | 59 (24-107) | 1048 (643-1470) |
| France | 41.7 (23.3-62) | 110.2 (87.6-134.0) | 4.9 (2.1-8.3) | 86.3 (52.6-120.3) | 8941 (4999-13,281) | 23,600 (18,757-28,711) | 1045 (450-1788) | 18,489 (11,269-25,776) |
| Germany | 22.9 (10.1-42.6) | 105.4 (75.6-141.7) | 15.2 (5.3-32.4) | 62.2 (36.8-90.8) | 4878 (2144-9077) | 22,447 (16,093-30,167) | 3239 (1118-6890) | 13,238 (7840-19,337) |
| Greece | 11.1 (5.8-18.0) | 11.4 (7.7-16.1) | 0.9 (0.2-2.5) | 3.6 (2.0-5.7) | 1850 (967-2998) | 1900 (1287-2679) | 145 (32-422) | 606 (340-940) |
| Hungary[a] | 2.6 (1.5-3.8) | 7.9 (6.3-9.6) | 0.5 (0.2-0.8) | 3.2 (2.0-4.5) | 218 (123-322) | 666 (530-809) | 39 (17-65) | 272 (166-380) |

| Country | Excess number of bed-days in thousands ($CI_{95}$) | | | | Excess costs in thousand Euros ($CI_{95}$) | | | |
|---|---|---|---|---|---|---|---|---|
| | MRSA | MSSA | G3CREC | G3CSEC | MRSA | MSSA | G3CREC | G3CSEC |
| Iceland[a] | 0.0 | 0.6 (0.4-0.7) | 0.0 (0.0-0.1) | 0.3 (0.2-0.4) | 0 | 164 (131-199) | 9 (4-15) | 86 (52-120) |
| Ireland[a] | 4.7 (2.6-6.9) | 7.1 (5.6-8.6) | 0.7 (0.3-1.2) | 4.8 (2.9-6.7) | 1367 (770-2023) | 2065 (1642-2507) | 203 (90-337) | 1397 (851-1949) |
| Israel | 5.3 (2.9-7.9) | 10.0 (7.8-12.3) | 4.4 (1.9-7.4) | 9.8 (6.0-13.8) | 662 (367-991) | 1252 (977-1545) | 553 (240-930) | 1228 (751-1721) |
| Italy | 24.7 (13.7-37.2) | 44.4 (34.5-55.0) | 9.1 (3.9-15.6) | 26.6 (16.1-37.5) | 4811 (2670-7252) | 8635 (6718-10,711) | 1770 (758-3046) | 5183 (3132-7310) |
| Latvia | 0.3 (0.1-0.4) | 2.7 (2.1-3.3) | 0.2 (0.1-0.3) | 0.4 (0.2-0.5) | 16 (8-27) | 162 (127-203) | 11 (4-21) | 23 (14-33) |
| Lithuania | 0.3 (0.2-0.5) | 3 (2.4-3.7) | 0.2 (0.1-0.4) | 1.0 (0.6-1.4) | 19 (10-32) | 180 (141-224) | 13 (5-24) | 61 (37-86) |
| Luxembourg[a] | 0.2 (0.1-0.3) | 0.8 (0.6-0.9) | 0.1 (0.0-0.1) | 0.8 (0.5-1.1) | 93 (52-138) | 328 (261-398) | 37 (16-61) | 319 (194-445) |
| Malta[a] | 0.5 (0.3-0.8) | 0.4 (0.3-0.5) | 0.1 (0.1-0.2) | 0.3 (0.2-0.4) | 51 (29-76) | 43 (34-53) | 12 (5-20) | 30 (18-41) |
| Netherlands | 0.3 (0.1-0.9) | 25.6 (20.0-31.6) | 1.6 (0.6-3.0) | 13.6 (8.2-19.1) | 78 (21-208) | 5971 (4662-7390) | 378 (150-711) | 3177 (1923-4467) |
| Norway | 0 | 11.0 (8.6-13.5) | 0.5 (0.2-0.9) | 8.1 (4.9-11.3) | 0 | 3865 (3033-4758) | 164 (65-306) | 2850 (1736-3996) |
| Poland | 3.0 (1.5-4.9) | 15.2 (11.6-19.5) | 0.5 (0.1-1.2) | 8 (4.8-11.4) | 195 (98-322) | 998 (758-1277) | 33 (9-81) | 523 (317-747) |
| Portugal | 18.4 (10.3-27.3) | 18.5 (14.6-22.6) | 3.4 (1.5-5.7) | 11.1 (6.7-15.5) | 2276 (1277-3383) | 2294 (1812-2803) | 423 (186-712) | 1378 (837-1922) |
| Romania | 1.3 (0.6-2.5) | 3.3 (2.1-4.8) | 1.8 (0.7-3.4) | 1.6 (0.9-2.4) | 57 (25-110) | 146 (96-213) | 79 (30-151) | 71 (40-107) |
| Slovenia[a] | 0.3 (0.2-0.5) | 3.4 (2.7-4.1) | 0.3 (0.1-0.5) | 2.4 (1.5-3.3) | 31 (17-46) | 319 (254-387) | 26 (12-43) | 226 (138-315) |
| Spain | 20.5 (11.4-30.8) | 57.1 (45.1-70.0) | 10.9 (4.8-18.5) | 52.9 (32.3-73.9) | 3529 (1964-5299) | 9816 (7749-12,030) | 1882 (825-3186) | 9097 (5553-12,704) |

| Country | Excess number of bed-days in thousands ($CI_{95}$) | | | | Excess costs in thousand Euros ($CI_{95}$) | | | |
|---|---|---|---|---|---|---|---|---|
| | MRSA | MSSA | G3CREC | G3CSEC | MRSA | MSSA | G3CREC | G3CSEC |
| Sweden | 0.2 (0.1-0.4) | 21.7 (17.3-26.3) | 0.7 (0.3-1.2) | 12.6 (7.7-17.6) | 58 (32-90) | 5432 (4323-6588) | 178 (79-297) | 3152 (1923-4398) |
| Turkey | 36.6 (20.5-55) | 65.8 (52.0-80.5) | 35.1 (15.4-59.0) | 19.2 (11.6-27.0) | 1705 (956-2560) | 3061 (2420-3748) | 1634 (719-2747) | 892 (540-1259) |
| UK[a] | 50.9 (28.7-75.4) | 105.5 (83.9-128.1) | 22.3 (9.9-36.9) | 59.7 (36.4-83.3) | 11,755 (6622-17,393) | 24,350 (19,362-29,557) | 5147 (2279-8525) | 13,782 (8398-19,233) |
| **Total** | 255.7 (142.9-375.9) | 691.4 (548.5-840) | 120.1 (52.3-198.3) | 426.2 (259.6-594.9) | 43,961 (23,885-67,786) | 128,701 (99,791-160,510) | 18,068 (7535-32,216) | 83,087 (50,337-117,131) |

Countries include all European Union member states (excluding Slovakia), both candidate countries (Croatia and Turkey), two EFTA countries (Iceland and Norway), and Israel.

[a] Total number as reported to EARSS or by national health authorities (UK), CI solely based on CI of the excess length of stay estimate from the clinical outcome studies; $CI_{95}$= 95% confidence interval; Rep. = Republic
